# Mitochondrial Dysfunction in Cardiac Disease: The Fort Fell

**DOI:** 10.3390/biom14121534

**Published:** 2024-11-29

**Authors:** Ioannis Paraskevaidis, Christos Kourek, Dimitrios Farmakis, Elias Tsougos

**Affiliations:** 1 Medical School of Athens, National and Kapodistrian University of Athens, 15772 Athens, Greece; iparask@med.uoa.gr (I.P.); farmakis.dimitrios@ucy.ac.cy (D.F.); 2 Department of Cardiology, Hygeia Hospital, 15123 Athens, Greece; cardio@tsougos.gr

**Keywords:** heart failure, mitochondria, cardiac disease, energy

## Abstract

Myocardial cells and the extracellular matrix achieve their functions through the availability of energy. In fact, the mechanical and electrical properties of the heart are heavily dependent on the balance between energy production and consumption. The energy produced is utilized in various forms, including kinetic, dynamic, and thermal energy. Although total energy remains nearly constant, the contribution of each form changes over time. Thermal energy increases, while dynamic and kinetic energy decrease, ultimately becoming insufficient to adequately support cardiac function. As a result, toxic byproducts, unfolded or misfolded proteins, free radicals, and other harmful substances accumulate within the myocardium. This leads to the failure of crucial processes such as myocardial contraction–relaxation coupling, ion exchange, cell growth, and regulation of apoptosis and necrosis. Consequently, both the micro- and macro-architecture of the heart are altered. Energy production and consumption depend on the heart’s metabolic resources and the functional state of the cardiac structure, including cardiomyocytes, non-cardiomyocyte cells, and their metabolic and energetic behavior. Mitochondria, which are intracellular organelles that produce more than 95% of ATP, play a critical role in fulfilling all these requirements. Therefore, it is essential to gain a deeper understanding of their anatomy, function, and homeostatic properties.

## 1. Introduction

The heart can be likened to a household where nearly everything depends on energy availability to meet the daily needs of each family member. Accordingly, myocardial cells and the extracellular matrix fulfill their roles thanks to the availability of energy. In fact, the mechanical and electrical properties of the heart are strongly dependent on maintaining a balance between energy production and consumption. Cardiac fuel serves as the master key for crucial processes such as contraction–relaxation coupling, ion exchange, cell growth, apoptosis, necrosis, and the maintenance of cardiac homeostasis. Interestingly, although the heart makes up only 0.5% of body weight, it consumes 8% of the body’s energy, and the ATP produced supports only a limited number of heartbeats. This forces the entire metabolic machinery to repeat the process every few seconds to meet the heart’s energetic demands [1,2]. On average, each heartbeat requires approximately 2 to 3 micromoles (μmol) of ATP per gram of heart tissue [3,4]. This translates to about 6 kg of ATP per day for the heart in a healthy adult, underscoring its high energy demand. Given that the heart beats around 100,000 times per day, this amounts to roughly 30 to 40 mg of ATP per heartbeat in a typical adult [3,4]. About 60–70% of this ATP is used for contractile functions, primarily supporting the cyclic interactions of actin and myosin filaments, while the remaining 30–40% is allocated to ion transport processes, such as calcium cycling, which is vital for muscle contraction and relaxation phases [3,4].

There are several forms of energy produced, including kinetic, dynamic, and thermal energy. Notably, under certain conditions such as aging and cardiac disease, while total energy remains constant, the contribution of each energy form changes. For example, in heart failure patients, thermal energy increases, while dynamic and kinetic energy decrease, making them insufficient to support cardiac function adequately [5]. Energy production and transfer within cardiac cells are impaired, as evidenced by a decrease in cellular ATP, phosphocreatine (PCr), and the PCr/ATP ratio, observed in both heart failure with reduced [6] and preserved left ventricular ejection fraction [7]. When this bioenergetic capacity reaches its limit, decompensation begins, leading to cardiac homeostatic imbalance. This is driven by the overactivation of the sympathetic nervous system, the renin–angiotensin–aldosterone system, inflammation, and other factors, resulting in a vicious cycle that leads to heart failure syndrome. The metabolic–energetic dysfunction promotes the accumulation of toxic products, including unfolded/misfolded proteins and free radicals, which contribute to changes in the heart’s micro- and macro-architecture, ultimately leading to cardiac remodeling [8,9,10]. Specifically, these alterations include i. misfolded proteins in the mitochondrial respiratory chain complexes which lead to inefficient ATP production and increase oxidative stress, also impairing electron transport [11], ii. dysfunction in heat shock proteins that signal a stress response that further exacerbates mitochondrial dysfunction [12,13], iii. alteration in mitochondrial dynamics proteins such as DRP1 (Dynamin-related protein 1) and mitofusins (MFN1/2), which are responsible for mitochondrial fission and fusion, leading to mitochondrial fragmentation and further bioenergetic and structural issues within cardiomyocytes [14,15], and iv. upregulation of BNIP3 and other apoptotic proteins due to oxidative stress, promoting cell death pathways, which contribute to cell loss in cardiac tissue and lead to maladaptive remodeling of the heart [16,17].

This bioenergetic capacity—energy production and consumption—depends on the heart’s metabolic resources and the functional status of both cardiomyocytes and non-cardiomyocyte cells, as well as their metabolic and energetic behavior. Understanding this behavior, especially the role of mitochondria—the master key in this process—requires further investigation.

## 2. Mitochondrial Dynamics

Mitochondria are intracellular organelles that produce more than 95% of ATP required by the whole body. Their normal structure, integrity, function, and homeostatic properties are crucial, as their improper anatomy and abnormal or altered function can lead to myocardial cell injury and death, contributing to the onset and progression of cardiac disease [1,18,19]. Mitochondria have their own DNA (mtDNA), which is circular in shape and encodes 13 protein subunits, while the majority of mitochondrial proteins are encoded by nuclear DNA and transported into the mitochondria via the mitochondrial membrane. Due to the limited protective mechanisms of mtDNA, it is prone to mutations that are responsible for many inherited cardiomyopathies [1]. However, the presence of mutated mtDNA in individuals often exceeds the incidence of myocardial diseases, acting as a dormant source for future diseases when mitochondrial mutations reach a certain threshold [20]. An example of a disease linked to mtDNA mutations reaching a pathogenic threshold is mitochondrial cardiomyopathy. This condition can develop when a critical proportion—often cited as about 60–90% mutant mtDNA in affected cells—accumulates, surpassing the cell’s ability to compensate [21,22]. This threshold can vary depending on the specific mutation and the tissue’s energy demands [21,22].

Interestingly, gene–gene and gene–environment interactions do not proportionally affect cardiac mitochondria, thanks to their powerful compensatory mechanisms, which provide resistance to external harmful events and protect against mitochondrial dysfunction and heart disease [19]. However, when mitochondria are severely affected, they reach a non-viable state that leads to harmful effects. Typically, mitochondria are only moderately affected, giving them time to compensate, restore homeostasis, and adjust their metabolic actions. If this compensatory process fails, heart diseases emerge and gradually worsen over time [23,24,25].

**Figure 1:**
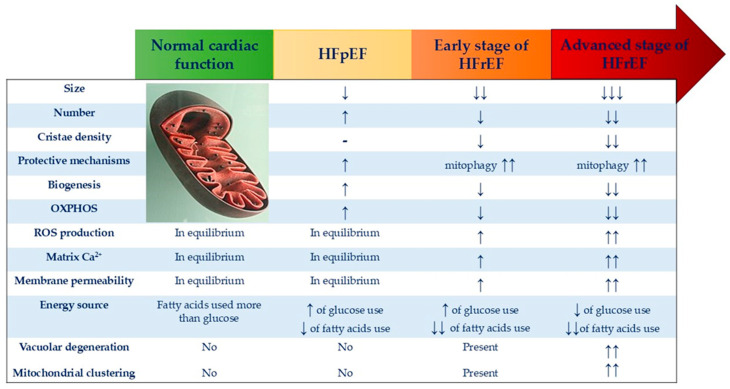
Changes in mitochondrial function and structure occur throughout the progression of heart failure syndrome. As heart failure advances, vacuolar degeneration of the mitochondria becomes evident, along with increased membrane permeability, altered biochemical substrate utilization, and heightened production of free radicals. Although defensive mechanisms increase in response to these changes, they eventually become insufficient to prevent the impending decompensation as the syndrome worsens. Ultimately, the heart’s defenses are overwhelmed, leading to failure. The fort fell. p: preserved, r: reduced, ↑: increase, ↓: decrease, ↑↑: bigger increase, ↓↓: bigger decrease, ↓↓↓ much bigger decrease.

In the early stages of heart failure syndrome, mitochondrial fission and fragmentation processes are activated. Mitochondrial fission and fragmentation could be measured through several advanced imaging and biochemical techniques including high-resolution fluorescence microscopy with mitochondrial staining, transmission electron microscopy (TEM), quantitative image analysis using specific software or specialized tools for mitochondrial morphology, Western blotting and immunostaining for fission/fusion proteins, and flow cytometry with specific mitochondrial stains [26,27,28]. As the syndrome progresses, there is a decrease in mitochondrial cristae density, the appearance of mitochondrial clusters, vacuolar degeneration, and calcium overload, all of which contribute to myocardial cell apoptosis and necrosis [25,29,30,31]. In response, mitochondrial defense mechanisms, particularly mitophagy, are upregulated to protect myocardial cells and the heart as a whole. This has been observed in both preserved and reduced ejection fraction heart failure, with mitophagy being more active in the latter [29,32,33,34]. However, while these protective mechanisms are initially beneficial, they become overwhelmed as the syndrome worsens, leading to mitochondrial dysfunction [34,35]. Additionally, the autophago-lysosomal system becomes dysfunctional [36,37], making mitochondria more vulnerable. As a result, heart failure of any cause continues to deteriorate, signaling the collapse of the heart’s defenses, as illustrated in Figure 1.

Mitophagy, a specialized form of autophagy, is the process by which damaged or dysfunctional mitochondria are selectively degraded to maintain cellular health, especially in energy-demanding cells like cardiomyocytes. PINK1 (PTEN-induced putative kinase 1) and Parkin play central roles in regulating mitophagy [38,39,40]. Under normal conditions, healthy mitochondria continually import and degrade PINK1, keeping Parkin, an E3 ubiquitin ligase, inactive in the cytosol [38,39,40]. However, when mitochondria become depolarized or damaged, PINK1 accumulates on the mitochondrial outer membrane and recruits Parkin to initiate mitophagy [38,39,40]. Parkin ubiquitinates various outer membrane proteins, signaling the cell’s autophagy machinery to engulf and degrade the impaired mitochondria [38,39,40]. This protective process is essential in cardiac pathologies, where mitochondrial stress and dysfunction contribute to disease progression, such as in heart failure. By clearing out defective mitochondria, mitophagy helps sustain energy production and reduces reactive oxygen species (ROS) accumulation, supporting cardiomyocyte survival and function under metabolic or oxidative stress [38,39,40]. In situations where mitophagy fails or is overwhelmed, as seen in many heart diseases, cells face an accumulation of dysfunctional mitochondria, leading to further energy deficits and cellular damage, ultimately accelerating disease progression [38,39,40].

**Figure 2:**
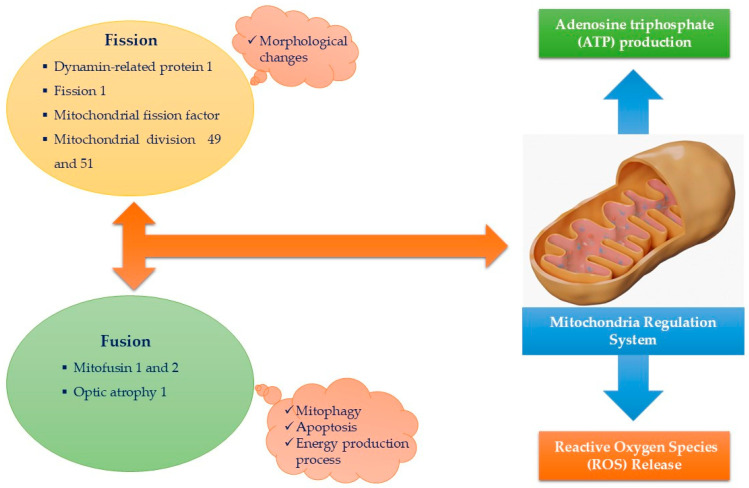
The primary functions of mitochondria are the production of adenosine triphosphate (ATP) and the release of reactive oxygen species (ROS). These functions are regulated by proteins involved in mitochondrial shaping, which are governed by the processes of fission and fusion, each controlled by specific proteins. Fission is associated with mitochondrial morphological changes, while fusion is linked to processes such as mitophagy, apoptosis, and energy production. Together, these systems ensure proper mitochondrial function and cellular homeostasis.

Mitochondria exhibit various shapes throughout the human body, influenced by the specific tissue and surrounding cellular environment. These factors affect mitochondrial structure, function, and behavior, leading to distinct mitochondrial subpopulations that respond differently to metabolic and energetic conditions, emphasizing their complexity, heterogeneity, and diversity. In the myocardium—the most metabolically active and mitochondria-rich organ—mitochondria play an essential role in regulating biogenesis, ion transport, and implementing protective mechanisms such as fusion, fission, and mitophagy [41,42] (Figure 2). This functionality is supported by the presence of three distinct mitochondrial subtypes within cardiomyocytes: (a) interfibrillar, (b) subsarcolemmal, and (c) perinuclear mitochondria. Each subtype displays unique shapes and specific responses to metabolic and pathophysiological changes. Interfibrillar mitochondria are oval, positioned in longitudinal rows between myofibrils, and demonstrate higher rates of oxidation. Subsarcolemmal mitochondria are involved in electrolyte and metabolite transport and enhance myocardial protection. Perinuclear mitochondria, spherical in shape, control nuclear function and regulate mitochondrial fusion and fission [41].

Mitochondrial respiratory chain complexes (I–IV), located within the inner mitochondrial membrane, assemble to form supercomplexes. These “respirasomes” (I + III2 + IV1) increase the efficiency of electron transfer, supporting mitochondrial and phospholipid functions (e.g., cardiolipin) to better meet the heart’s energy demands. Furthermore, these supercomplexes aid in reducing free radical production, thereby safeguarding against mitochondrial dysfunction [43,44]. As noted, the primary role of mitochondria is energy production, with nearly 90% of ATP generated being utilized to support contraction–relaxation coupling. The separation and reassembly of actin and myosin are highly energy-dependent processes directly linked to mitochondrial ATP production. Similarly, ion exchange, particularly calcium (Ca^2+^) release and sequestration, requires significant energy, provided by mitochondria.

Mitochondria must rapidly respond to the body’s energy demands, especially during periods of high exertion, such as intense exercise, or under pathological conditions like myocardial ischemia, hypertension, cardiac hypertrophy, or heart failure. To adapt, mitochondria are capable of self-protection, interconnection, morphological changes, and movement within cells, even crossing cell boundaries [45]. These adaptations may occur under normal physiological conditions or in response to clinical scenarios such as myocardial ischemia or heart failure. When mitochondria become mutated or dysfunctional, energy production declines, while harmful substances—such as free radicals, heat shock proteins, and unfolded or misfolded proteins—accumulate, contributing to the onset and progression of cardiac diseases.

These findings underscore the necessity for mitochondria to have self-protective mechanisms to prevent dysfunction and maintain the cell’s energetic, metabolic, and homeostatic balance. Mitochondrial morphology and function adapt to varying environments, activating self-defense processes essential for cell survival [41] (Figure 2). These processes are regulated by specific proteins, including the guanosine triphosphate (GTP) hydrolase enzyme family, mitochondrial fission and fusion proteins, and mitochondrial dynamics proteins 49 and 51, which facilitate continuous adaptation of mitochondrial shape and function, promote genetic material exchange, and ensure optimal performance [41,46]. Although the precise mechanisms by which mitochondria receive genetic material from other cells are not fully understood, they suggest an intercellular communication pathway that helps prevent mitochondrial malfunction [45].

Mitochondrial dynamics, involving fission, fusion, and mitophagy, play critical roles in the development and progression of HF. Fission, driven largely by the protein Drp1, promotes the division of mitochondria, a process necessary for removing damaged sections but which can lead to excessive fragmentation when dysregulated, as seen in HF [47,48,49,50,51,52]. This fragmentation reduces the efficiency of the mitochondrial network, increasing ROS production and decreasing ATP output, which are detrimental to cardiomyocytes. Fusion, regulated by proteins such as mitofusins (Mfn1 and Mfn2) and OPA1, enables damaged mitochondria to merge with healthy ones, diluting mutations and sharing metabolic resources [47,48,49,50,51,52]. Fusion supports a more interconnected mitochondrial network that resists stress, but impaired fusion leads to energy deficits and decreased mitochondrial resilience, especially impactful in HF with reduced ejection fraction (HFrEF). In this type of HF, mitochondrial dysfunction is more severe, and an upregulation in mitophagy—the selective degradation of damaged mitochondria via PINK1/Parkin pathways—attempts to clear failing mitochondria to maintain cellular function [47,48,49,50,51,52]. However, in both preserved ejection fraction (HFpEF) and HFrEF, these mechanisms eventually become overwhelmed. In HFpEF, where mitochondrial dynamics are disrupted but less severely than in HFrEF, compensatory mechanisms like mitophagy help sustain function longer. Thus, the balance and integrity of mitochondrial dynamics are essential to HF progression, with each subtype experiencing distinct mitochondrial stress responses, influencing therapeutic targets and disease management [47,48,49,50,51,52].

Three distinct modes of intercellular mitochondrial transport have been proposed: (a) tunneling nanotubes (TNTs), (b) membrane extracellular vesicles (EVs), and (c) gap junctions (GJCs) [53]. TNTs are the primary means of mitochondrial transport, forming quickly from mitochondrial membrane protrusions and comprising F-actin and transport proteins [54]. Membrane microvesicles, also known as extracellular vesicles (EVs), are heterogeneous structures released from the intracellular to the extracellular environment. Smaller EVs carry exosomes, small RNAs, genomic DNA, and mtDNA, while larger EVs may contain entire mitochondria [45,55,56]. Their main functions include eliminating abnormal proteins and facilitating intercellular communication, especially within the nervous system [57,58]. GJCs act as transport channels for various substances, including nutrients, metabolites, and mitochondria [59], and appear to play a role in the intercellular transfer of reactive oxygen species (ROS) [42,60].

Although mitochondrial structural changes have been associated with various pathologies, this understanding has not yet been fully integrated into routine clinical practice [42,61]. Certain mitochondrial phenotypes, such as donut-like or ellipsoid shapes, may represent defensive adaptations to harmful stimuli [61,62,63], potentially affecting key mitochondrial protective mechanisms like fission and fusion. Given the existence of distinct mitochondrial subpopulations and the roles of specific proteins—such as mitochondrial fission factor (MFF), mitochondrial division proteins (Drp1, 49, and 51), and fusion proteins (mitofusin 1 and 2) (Figure 2)—altered mitochondrial morphology could serve as an early indicator of disease, and quantifying these factors may aid in clinical diagnosis. Furthermore, studying mtDNA heteroplasmy (the presence of different alleles within the same patient) [64] could offer valuable insights into mitochondrial abnormalities and help identify potential future complications at an earlier stage.

Proteins such as dynamin-related protein 1 (Drp1), mitofusins (Mfn1 and Mfn2), and optic atrophy protein 1 (OPA1) are central to the regulation of mitochondrial dynamics, directly influencing mitochondrial shape, structure, and network integrity, all of which are crucial for cardiac health [65,66]. These proteins modulate two key processes: mitochondrial fission (division) and fusion (joining), both of which ensure optimal mitochondrial function and energy production, especially in energy-demanding tissues like the heart [65,66]. Their balanced regulation is essential for maintaining mitochondrial integrity and function. Alterations in their expression levels disrupt mitochondrial dynamics, leading to fragmented or dysfunctional mitochondria, energy deficits, and increased oxidative stress—all of which can contribute to the onset and progression of cardiac diseases [65,66].

Drp1 is essential for mitochondrial fission, enabling mitochondria to divide and remove damaged sections. Upregulation of Drp1 can lead to excessive mitochondrial fragmentation, which, while helpful under certain stress conditions, may impair mitochondrial network continuity and reduce overall efficiency in ATP production if sustained [65,67]. In heart cells, excessive Drp1 activity can lead to a fragmented mitochondrial network, which disrupts energy supply, increases ROS production, and may contribute to cardiomyocyte apoptosis, exacerbating heart failure and other cardiac pathologies [65,67].

Mitofusins (Mfn1 and Mfn2) are critical for mitochondrial fusion, allowing mitochondria to merge and share contents, including DNA and metabolic resources, which helps maintain a healthy mitochondrial population [50,68]. The upregulation of Mfn1 and Mfn2 promotes a more interconnected mitochondrial network, supporting efficient ATP production and resistance to cellular stress [50,68]. However, downregulation or mutations in mitofusins have been linked to disrupted mitochondrial fusion, leading to a fragmented and less efficient network. In cardiomyocytes, reduced Mfn activity can impair calcium handling, fuel metabolism, and overall contractile function, potentially leading to cardiac hypertrophy and heart failure [50,68].

OPA1 plays a dual role in inner mitochondrial membrane fusion and maintaining cristae structure, which is vital for efficient ATP production within the mitochondria. Upregulation of OPA1 supports mitochondrial stability and helps preserve cristae integrity, enhancing the efficiency of electron transport chain complexes [69,70]. Conversely, OPA1 downregulation destabilizes cristae structure, impairing ATP production and increasing susceptibility to apoptosis [69,70]. In myocardial cells, this can reduce the energy available for contraction–relaxation cycles, leading to contractile dysfunction and contributing to the progression of heart disease [69,70].

## 3. Mitochondria: A ‘Socialized’ Organelle

Cellular organelles are interconnected, functioning not as isolated units but as part of a coordinated system responsive to the cell’s needs [71]. The anatomical and functional communication between organelles—such as the endoplasmic reticulum (ER), mitochondria, nucleus, plasma membrane, and Golgi apparatus—is well established, underscoring their crucial role in maintaining homeostasis within the human body [72,73]. Mitochondria, for instance, are not formed de novo and lack certain biosynthetic capabilities, such as synthesizing phosphatidylcholine, phosphatidylinositol, sterols, and sphingolipids [73]. Consequently, their functions are closely dependent on interactions with other organelles [71,73].

Mitochondria are often regarded as the most “socialized” organelle due to their extensive interconnections with various cellular components. Their defensive mechanisms—such as fusion, fission, and mitophagy—rely heavily on effective communication with other organelles. Notably, mitochondria interact with lysosomes [74,75], peroxisomes [76], and lipid droplets [77], facilitating optimal cellular homeostasis and function. Of particular significance is the continuous communication between the ER and mitochondria. This interaction is essential because mitochondrial functions such as oxidative phosphorylation, ATP production, and Ca^2+^ exchange and buffering are dependent on efficient lipid and Ca^2+^ transport from the ER [18,73].

### 3.1. The Endoplasmic Reticulum (ER) and Mitochondria: Interconnected Organelles in Cardiovascular Health

The ER is involved in numerous cellular processes, including secretion, protein folding, ion homeostasis, and lipid biosynthesis, and it communicates with other cellular organelles to regulate these activities. In cardiovascular diseases, factors such as ischemia, pulmonary and arterial hypertension, and metabolic disorders can disrupt normal ER function, leading to homeostatic imbalances characterized by increased free radical production and accumulation of misfolded proteins. This disruption impairs communication between the ER and other cardiomyocyte organelles [78], promoting processes like apoptosis and necrosis. Specifically, the connection between the ER and mitochondria, via mitochondria-associated membranes (MAMs), is essential for proper mitochondrial function, including cellular metabolism, ion homeostasis, and inflammation regulation. The ultrastructural organization between these two organelles governs numerous critical cellular processes [72] and plays a pivotal role in cardiovascular remodeling and the progression of various cardiovascular diseases [79,80].

In healthy myocardial cells, MAMs ensure efficient calcium transfer from the ER to mitochondria, support lipid synthesis and trafficking, and facilitate communication that aligns mitochondrial function with cellular metabolic demands [81,82]. Specifically, one of the primary roles of MAMs is to mediate rapid and controlled transfer of Ca^2+^ from the ER to mitochondria [81,82]. This process is regulated by specific proteins at MAM sites, such as the inositol 1,4,5-trisphosphate receptors (IP_3_Rs) on the ER membrane and the voltage-dependent anion channel (VDAC) on the outer mitochondrial membrane [81,82]. MAMs also facilitate the transfer of lipids, such as phosphatidylserine, from the ER to mitochondria, where it is converted into phosphatidylethanolamine, a critical component of mitochondrial membranes. This lipid exchange supports membrane integrity, promotes mitochondrial dynamics, and aids in the synthesis of cardiolipin, a lipid essential for the optimal function of respiratory chain complexes [83,84]. Efficient lipid trafficking ensures that the mitochondria can maintain their structure and support the high levels of energy production needed for cardiac function [83,84]. Finally, MAMs act as signaling hubs, coordinating responses to cellular stress and helping to regulate apoptosis. They play a crucial role in activating stress responses that protect cardiomyocytes from injury under conditions such as ischemia or oxidative stress [85].

Disruption of ER–mitochondria communication can lead to redox imbalances, ER stress, mitochondrial injury, calcium (Ca^2+^) homeostasis imbalance, energy depletion, and programmed cell death. A detailed assessment of ER–mitochondria communication and the impact of its disruption on cellular function could be measured through biochemical and imaging techniques such as fluorescence resonance energy transfer (FRET) or bioluminescence resonance energy transfer (BRET), transmission electron microscopy, proximity ligation assay (PLA), Western blotting for MAMs, immunoprecipitation for protein complexes and functional assays for redox and calcium homeostasis [86,87,88]. Consequently, myocardial contraction–relaxation coupling and vascular smooth muscle cell differentiation are impaired. Damaged or dysfunctional mitochondria produce large amounts of reactive oxygen species (ROS), which accumulate within the cell, exacerbating myocardial injury. In heart failure patients, elevated levels of free iron within mitochondria—a critical ion in free radical production through Fenton chemistry—further contribute to oxidative stress [89]. Whether the ER or mitochondria are initially affected, the resulting loss of homeostasis and communication between these organelles leads to incomplete cardiomyocyte repair, oxidative stress imbalances [90], Ca^2+^ dysregulation [91], abnormal lipid metabolism [92], insufficient energy production [72], and activation of mitochondria-associated membranes (MAMs), which contribute to inflammasome formation and inflammatory processes [17,93]. When mitochondrial and ER structure and function are severely compromised, protective mechanisms become overwhelmed, releasing toxic substances such as nuclear and mitochondrial DNA into the cytosol [94], marking the onset and progression of cardiovascular diseases.

**Figure 3:**
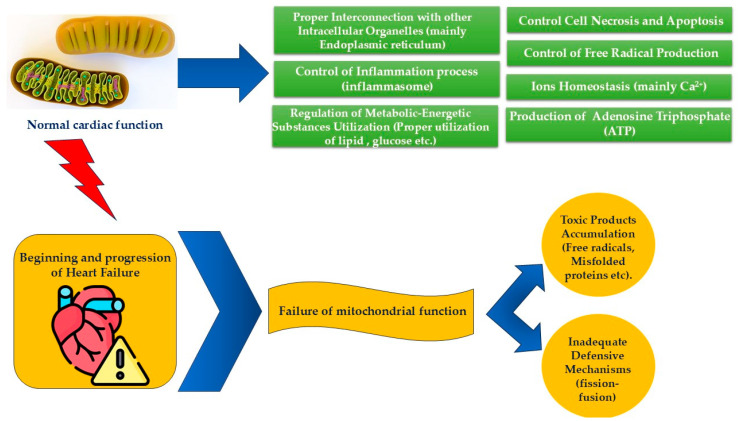
Mitochondrial function is crucially dependent on several factors: the efficient utilization of metabolic resources, proper communication with other organelles, regulated ion exchange, minimal accumulation of free radicals and harmful byproducts, and the ability to control cell necrosis and apoptosis. Protective mechanisms such as mitochondrial fission and fusion play an essential role in maintaining mitochondrial integrity and function. These processes help adapt to cellular stress and damage, ensuring the organelles continue to operate normally. However, when these protective mechanisms fail—whether due to a breakdown in homeostasis or malfunctioning of the fission and fusion processes—mitochondrial dysfunction ensues. This dysfunction marks the onset of heart failure syndrome, which progressively worsens over time as the mitochondria can no longer sustain normal cellular function.

Cardiovascular diseases are frequently associated with cardiac remodeling, involving abnormal structural and functional changes within the cardiovascular system [95]. These alterations result from abnormal responses to stimuli and involve inflammation, defects in autophagy, impaired gene transcription, energy metabolism deficiencies, increased oxidative stress, ion homeostasis imbalances, apoptosis, and necrosis [96,97,98,99,100,101]. A significant factor in these changes is the uncoupling of sarcoplasmic/endoplasmic reticulum-mitochondria interactions [102,103], while proper coupling is crucial for maintaining cellular stability [104]. Cardiomyocytes, which are highly energy-dependent, rely on the connection between these two organelles to regulate key processes such as Ca^2+^ buffering and transport [95]. Mitochondria act as major calcium reservoirs [105] and play a central role in biochemical processes such as lipid metabolism and calcium signaling [106]. Similarly, the ER serves as the primary regulator of Ca^2+^ homeostasis [107] and a critical site for protein and lipid biosynthesis [108]. Therefore, communication between these organelles is essential for two main cardiomyocyte functions: (a) Ca^2+^ buffering and transport, vital for contraction–relaxation coupling, and (b) oxidative phosphorylation, which meets the energetic demands of the myocardium (Figure 2 and Figure 3). Dysfunctional ER–mitochondrial interactions contribute to the development of various cardiovascular diseases, including cardiac hypertrophy [109], heart failure, cardiomyopathy [110], ischemic heart disease [111], and arrhythmias [112].

Calcium is a critical ion for regulating mitochondrial redox processes and energy production. During cardiomyocyte contraction, Ca^2+^ is transferred from the ER and cytoplasm to mitochondria, activating functions essential for maintaining cardiomyocyte bioenergetics [95]. However, uncontrolled Ca^2+^ accumulation can severely impair mitochondrial function, leading to a loss of cellular homeostasis, activation of the mitochondrial apoptotic pathway, increased inflammation, and ultimately the onset and progression of heart failure (Figure 3). Mitochondrial dysfunction is also observed in patients with renal insufficiency, insulin resistance, and other comorbidities frequently associated with heart failure, underscoring the critical role that mitochondria play in overall human homeostasis and disease progression [1].

#### Mitochondria and Quadruple Therapy in Heart Failure Patients

In heart failure, the heart’s energy demands are significantly impaired by mitochondrial dysfunction, leading to poor contractility, increased ROS, and altered cell survival. Moreover, the pathophysiological basis of heart failure syndrome, regardless of its cause, lies in the treatment of the hyperactivation of the renin–angiotensin–aldosterone system (RAAS) and the sympathetic nervous system. This hyperactivation leads to metabolic imbalance, excessive free radical production, and the activation of inflammatory processes. Consequently, quadruple therapy for heart failure aims to block this hyperactivity to reduce myocardial oxygen consumption, reprogram the altered metabolic remodeling, and address the energetic needs of the heart [1,113,114,115,116]. By targeting mitochondrial dysfunction on multiple levels, these therapies collectively improve the energy state, reduce oxidative damage, and support mitochondrial quality control, translating to enhanced cardiac function and improved clinical outcomes.

Beta-blockers primarily reduce sympathetic overdrive, mitigating ROS production and preventing mitochondrial calcium overload. Most specifically, beta-blockers, such as carvedilol and metoprolol, reduce sympathetic nervous system (SNS) activation [117,118,119,120]. By blocking beta-adrenergic receptors, beta-blockers reduce heart rate, myocardial oxygen demand, and workload on the heart, which indirectly benefits mitochondrial function. They help with the reduction in the excessive catecholamine stimulation that increases mitochondrial ROS production, thus indirectly lowering ROS and thereby protecting mitochondrial integrity and function [117,118,119,120]. Moreover, they inhibit mitochondrial calcium overload caused by SNS activation, promoting thus, stable mitochondrial function and reducing the likelihood of mitochondrial permeability transition pore (mPTP) opening, a key driver of apoptosis [117,118,119,120]. Finally, carvedilol, has been shown to upregulate peroxisome proliferator-activated receptor-gamma coactivator-1α (PGC-1α), a master regulator of mitochondrial biogenesis, enhancing mitochondrial number and function, and potentially improving cardiac energy metabolism [117,118,119,120].

RAAS inhibitors, including angiotensin-converting enzyme (ACE) inhibitors and angiotensin receptor blockers (ARBs), decrease the adverse effects of angiotensin II and aldosterone, which are major contributors to cardiac remodeling, fibrosis, and cellular dysfunction in heart failure. They also alleviate oxidative stress, balance mitochondrial dynamics, and improve ATP efficiency [1,121,122,123,124,125]. Specifically, angiotensin II upregulates NADPH oxidase, promotes mitochondrial fission (fragmentation) and disrupts mitochondrial fusion. By blocking angiotensin II, RAAS inhibitors reduce ROS production, alleviating mitochondrial oxidative stress, and improve the balance between fission and fusion, which is crucial for maintaining healthy mitochondrial networks, supporting mitochondrial quality control and optimizing ATP production [1,121,122,123,124,125]. Additionally, decreased inflammation from RAAS inhibition helps reduce damage to mitochondrial membranes and proteins, preserving mitochondrial function [1,121,122,123,124,125]. Finally, RAAS inhibitors help alleviate the strain on mitochondrial ATP production through the reduction in the afterload and myocardial oxygen consumption [1,121,122,123,124,125]. Some studies also suggest RAAS inhibition can directly enhance mitochondrial oxidative phosphorylation efficiency, improving cellular energy availability [1,121,122,123,124,125].

Similarly, SGLT2 inhibitors positively influence metabolic and mitochondrial function [126,127,128], improving mitochondrial energetics and meeting the myocardial fuel demands [129]. SGLT2 inhibitors promote a shift toward ketone utilization, a more energetically efficient fuel compared to glucose and fatty acids, and enhance autophagy and mitochondrial resilience [130]. This reduces mitochondrial workload and oxidative stress while increasing ATP production efficiency, benefiting failing myocardial cells with compromised energy production. Moreover, they reduce inflammation and oxidative stress by mechanisms that may include reduced Na^+^ overload in myocardial cells, preserving thus, mitochondrial DNA, proteins, and membranes, and supporting mitochondrial function. Indeed, there are studies suggesting that SGLT2 inhibitors may enhance autophagy, which aids in the removal of damaged mitochondria and enhances mitochondrial turnover [131,132,133]. This dynamic regulation of mitochondrial quality helps maintain a healthy pool of mitochondria, thus improving cellular resilience in heart failure [131,132,133].

Neprilysin inhibitors, combined with ARBs in drugs like sacubitril/valsartan, enhance natriuretic peptide levels, counteracting the maladaptive neurohormonal effects in heart failure. Natriuretic peptides promote NO production, which has protective effects on mitochondria by reducing oxidative stress and improving endothelial function [134,135,136]. NO can inhibit excessive mitochondrial ROS production and stabilize mitochondrial function. Neprilysin inhibition also reduces the breakdown of natriuretic peptides, which promotes vasodilation, natriuresis, and reduces cardiac afterload. These effects decrease myocardial oxygen demand and ROS production, protecting mitochondrial integrity in the myocardium ana leading to reduction in mitochondrial oxidative stress [134,135,136]. Finally, neprilysin inhibitors present beneficial effect on the modulation of mitochondrial biogenesis and cell survival pathways [134,135,136]. Although the effects on mitochondrial biogenesis are not fully understood, these drugs have been observed to influence signaling pathways associated with cell survival, such as cyclic GMP (cGMP) pathways. Increased cGMP might support mitochondrial health indirectly by reducing apoptotic signaling and enhancing cellular stress resistance in heart failure [134,135,136].

Mitochondrial-targeted therapies hold promise not only for heart failure but also for other cardiovascular diseases where mitochondrial dysfunction plays a pivotal role, such as ischemic heart disease and cardiomyopathies. In ischemic heart disease, mitochondria experience oxidative damage due to fluctuating oxygen levels, leading to bioenergetic deficits and increased cell death; thus, treatments aimed at enhancing mitochondrial resilience could reduce ischemic injury [137]. In cardiomyopathies, where genetic mutations or acquired factors disrupt mitochondrial DNA and dynamics, therapies targeting mitochondrial function and integrity may prevent progression and improve cardiac outcomes [138,139]. Quadruple therapy’s impact on mitochondrial health underscores its potential not only as a therapeutic standard for heart failure but also as a foundation for further advances in mitochondrial-targeted therapies across cardiovascular diseases. By incorporating mitochondrial-protective strategies into broader cardiovascular care, these treatments could enhance cell survival, reduce ROS, and improve energy efficiency, addressing underlying mechanisms that drive a range of heart diseases [137,138,140,141,142,143].

However, despite the application of the recommended quadruple therapy for heart failure and other cardiovascular diseases, morbidity and mortality rates remain high, indicating that something is still lacking. Therefore, further scientific research is necessary to better understand the metabolic and energetic status of myocardial cells, with a particular focus on the interconnection and function of mitochondria and the endoplasmic reticulum (ER).

### 3.2. Unusual Location of Mitochondria

Another crucial area warranting further investigation is the emergence of cell-free mitochondria and mitochondrial DNA (mtDNA) circulating in the blood, released from various cells in response to stress, injury, or disease [144]. Although reports on their functionality are somewhat conflicting, it appears that these cell-free mitochondria may lack energetic activity [145]. Their presence in the bloodstream raises several pertinent questions that require in-depth exploration:

1. What is the significance of cell-free mitochondria in healthy versus diseased individuals? Understanding the role these particles play could provide valuable insights into cellular health and disease mechanisms.
2. What is the source of origin for these circulating mitochondria? Identifying the specific cells or tissues from which they are released could help clarify their role in various physiological and pathological contexts.
3. Can circulating mitochondria serve as therapeutic targets? If these mitochondria are found to influence disease progression, they could potentially be targeted in treatment strategies.
4. How do cells and tissues react to the presence of cell-free mitochondria in the bloodstream? Since these particles may represent non-self substances, it is essential to investigate the protective responses activated by individual cells and tissues to mitigate any harmful effects.

Interestingly, high levels of circulating cell-free mtDNA have been observed in various clinical contexts, including diabetes, cancer, and myocardial infarction, suggesting their potential as prognostic biomarkers [45,146]. Notably, elevated levels of cell-free mtDNA are not limited to specific cardiovascular diseases but are also associated with cell necrosis, acute respiratory distress syndrome, tumors, and inflammation of various origins [147,148].

In conclusion, the presence and implications of cell-free mitochondria and mtDNA in the bloodstream represent an open area of research with significant potential. Understanding their biological significance could lead to advancements in the diagnosis, prognosis, and treatment of a range of diseases, ultimately contributing to a more comprehensive understanding of cellular health and disease management.

## 4. Identify Mitochondria Dysfunction: Imaging Techniques and Biomarkers

Although mitochondria play a central role in several cardiac diseases, their detection through imaging techniques and/or blood sample analysis is not yet well established. Moreover, some available methods do not serve as optimal identifiers, are expensive, and therefore are not commonly used in everyday clinical practice. Additionally, it must be considered that of the total energy consumed by the heart, only 25% is used for mechanical purposes, while the remaining portion is allocated to non-mechanical processes such as metabolism and heat production [149,150]. These calculations were performed through invasive techniques via calculating the input energy by measuring coronary sinus blood flow and the oxygen content difference between arterial and venous blood, and advanced non-invasive imaging methods, such as positron emission tomography (PET) with carbon-11-labeled acetate or oxygen-15 tracers, and magnetic resonance spectroscopy (MRS) using phosphorus-31 [150,151,152].

Regarding imaging techniques, there are two approaches to indirectly calculate mitochondrial energy production capacity: invasive and non-invasive techniques. In the invasive technique, the input energy is measured by calculating the coronary sinus blood flow multiplied by the arteriovenous oxygen content difference, while the output energy can be assessed using the pressure–volume loop. Non-invasive techniques include PET, cardiovascular MRS [153], and the identification of metabolic disturbances in plasma [154].

For PET imaging, carbon-11-labeled acetate (11C-acetate) and oxygen-15-labeled molecular oxygen (15O2) tracers have been employed [150]. However, these tracers present several drawbacks, limiting their application. Phosphorus-31 (31P) magnetic resonance spectroscopy (MRS) can measure endogenous cardiac high-energy phosphate metabolites, creatine kinase (CK) flux [155,156,157], and other markers, demonstrating the mitochondrial energetic capacity [158,159,160].

PET and MRS offer unique advantages and limitations in assessing mitochondrial function. PET is highly sensitive and can quantify metabolic activity in real-time, allowing it to detect even subtle changes in mitochondrial energy production. It uses tracers, such as ^11^C-acetate and ^15^O_2_, to measure oxygen consumption and other metabolic processes, making it a powerful tool for studying mitochondrial efficiency and ATP generation. However, PET’s use of radioactive tracers, high cost, and limited availability can restrict its application, especially in routine clinical settings [161]. MRS, on the other hand, is non-invasive and does not rely on radiation, making it safer for repeated assessments. It can evaluate high-energy phosphate metabolites like ATP and phosphocreatine directly in the myocardium, offering valuable insights into mitochondrial bioenergetics [162,163]. Despite these benefits, MRS has lower sensitivity and resolution compared to PET, making it challenging to capture rapid metabolic changes or subtle mitochondrial dysfunctions [162,163]. Furthermore, MRS requires specialized equipment and expertise, which can limit its accessibility and clinical feasibility. Both methods provide valuable data, yet their limitations underscore the need for complementary approaches to obtain a comprehensive understanding of mitochondrial health.

In heart failure patients, mitochondrial function is impaired, biochemical processes are disrupted, and abnormal substances are utilized. This dysfunction can be detected using metabolomics; however, the source of these abnormal substances is unclear, reducing the accuracy of the technique [164,165]. While cardiovascular magnetic resonance spectroscopy holds promise, its use is limited due to inherent challenges [166].

Several other biomarkers have been explored, but none have shown the potential to provide robust information regarding mitochondrial function. Biomarkers such as lactate, pyruvate, the lactate-to-pyruvate ratio, and creatine phosphokinase have been used, but all show low specificity and sensitivity in detecting mitochondrial deficiency. Additionally, newer proposed biomarkers, such as growth differentiation factor 15 (GDF-15) and fibroblast growth factor 21 (FGF-21), are of interest but currently show limited diagnostic power [149].

GDF-15 and FGF-21 have been explored as potential biomarkers for mitochondrial dysfunction due to their roles in cellular stress response and metabolic regulation, particularly under conditions like oxidative stress and inflammation [167,168,169]. Both proteins are upregulated in response to mitochondrial stress, making them appealing candidates for monitoring mitochondrial health in pathologies such as heart failure. GDF-15, for instance, is produced in response to mitochondrial damage and is associated with oxidative stress, while FGF-21 is involved in energy metabolism and is elevated under metabolic stress conditions, such as impaired fatty acid oxidation [167,168,169]. Recent advancements in biomarker research are addressing these limitations by focusing on more specific markers of mitochondrial processes, such as mitochondrial DNA (mtDNA) release or specific metabolites like acylcarnitines that reflect mitochondrial activity more directly [170,171]. Challenges ahead include improving specificity and sensitivity while also identifying markers that reflect early mitochondrial dysfunction before significant cellular damage occurs. Techniques like metabolomics and multi-omics integration are being leveraged to identify complex biomarker patterns, paving the way for more precise and disease-specific mitochondrial biomarkers in the future [170,171].

## 5. Strategies to Keep Mitochondrial Structural and Functional Integrity

Two strategies exist to protect mitochondrial integrity: non-pharmacological and pharmacological approaches. The non-pharmacological approach includes exercise training and lifestyle modifications. While definitive conclusions are still lacking [41], evidence suggests that regular exercise promotes beneficial changes in mitochondrial function and metabolism [172,173], as well as in the activity of mitochondrial fusion and fission proteins [174], thereby demonstrating a cardioprotective effect [175,176]. Remarkably, even a few days of endurance exercise training can provide protection to mitochondria against ischemia–reperfusion injury [177]. The protective effects of endurance exercise on mitochondria, especially in the context of ischemia–reperfusion injury, are measured through various physiological, biochemical, and imaging methods including measurement of ROS levels, lipid peroxidation, and antioxidant enzyme activities in cardiac or skeletal muscle tissue, measurement of mitochondrial respiration and ATP production, calcium retention capacity (CRC), staining techniques, such as TUNEL (for apoptosis) or staining for necrotic markers, as well as changes in the expression of mitochondrial protective proteins in response to exercise, such as PGC-1α (a regulator of mitochondrial biogenesis), mitochondrial fusion proteins (e.g., MFN2, OPA1), and antioxidant enzymes through Western blotting, PCR, and immunohistochemistry [178,179,180,181]. Typical exercises that induce these protective effects include moderate-intensity endurance activities such as running, cycling, or swimming [178,179,180,181]. Similarly, although not fully proven, lifestyle habits, particularly calorie restriction, have been proposed to improve cardiac dysfunction by better controlling cardiac fibrosis, inflammation, and mitochondrial defense mechanisms [182,183].

In terms of pharmacological intervention, the current optimal medical treatment for heart failure, known as quadruple therapy, includes mechanisms aimed at conserving energy to restore mitochondrial function. However, despite the use of these treatments, morbidity and mortality rates remain high. Consequently, new medications are being explored to refine and enhance a truly optimal treatment approach. Various agents targeting metabolism (fatty acids, glucose) and antioxidants have been proposed [23,184]. For example, the modulation of peroxisome proliferator-activated receptor-α agonists and L-carnitine may improve left ventricular function and prevent myocardial fibrosis [185,186]. Likewise, SGLT2 inhibitors aid in restoring biochemical substrate utilization (fatty acid oxidation/glycolysis) and enhancing mitochondrial energetics [187]. Additionally, metformin, thiazolidinediones, and statins indirectly activate AMPK, thereby promoting mitochondrial biogenesis [188].

The use of sacubitril/valsartan increases levels of natriuretic peptides, particularly αANP [189], restores the ratio of the inner mitochondrial membrane (IMM) to the outer mitochondrial membrane (OMM), reduces ROS levels, and enhances autophagy, thereby exerting cardioprotective effects. While antioxidant drugs present a promising option, their results have been inconsistent. For instance, Coenzyme Q10 improved ejection fraction in one study [190], but showed no benefit in another [191].

Other pharmacological interventions, such as mitochondrial pyruvate carriers [192], mitochondrial permeability transition pore (mPTP) inhibitors like cyclosporine A [193], and various other agents [194,195,196,197,198], have been investigated, yet none have consistently demonstrated conclusive results. Some studies targeting mitochondrial fusion and fission mechanisms have shown improvements in mitochondrial function [68,199,200,201,202,203], while others have not [204,205,206,207,208,209]. Thus, this area of research clearly warrants further investigation.

## 6. Future Directions

The scientific community’s interest in mitochondrial structure and function is growing rapidly. However, there remains a lack of sufficient and robust data to accurately identify malfunctioning mitochondria. Despite advancements in this field, many questions remain unanswered and require further exploration. The diversity of mitochondrial phenotypes may aid in identifying various diseases, including cardiovascular conditions [210]. Alterations in key functional sites could reflect changes in mitochondrial energetic status and may potentially indicate early disease onset. The multi-scale mitochondrial configurations observed across different cell types are not yet fully understood, but they may represent an important step forward in this research [63].

In addition, little is known about the depletion or alteration of mitochondrial RNA (mtRNA), which could impact mitochondrial defense mechanisms and other vital functions [211]. Depletion of mtRNA, or other events that disrupt these protective mechanisms, could compromise mitochondrial defense [212]. Furthermore, do mitochondria-associated membranes (MAMs) play a preventive role in cardiovascular diseases? Could everyday habits influence MAMs and contribute to the progression of cardiovascular disease [213]?

In any case, our knowledge remains limited, and further efforts are essential to better understand this “energy factory,” especially within myocardial mitochondria. Could artificial intelligence assist in advancing this understanding? That question also remains open for future investigation [214].

## 7. Conclusions

Significant progress has been made over time in understanding heart failure syndrome. Among the key contributors to this condition are mitochondria—vital organelles influenced by risk factors, comorbidities, and other multifactorial mechanisms, which together contribute to the syndrome’s progression. Mitochondrial dysfunction in heart failure is marked by a decline in bioenergetic efficiency, reduced energy production, altered ion transport, increased free radical generation, and the production of misfolded proteins. This dysfunction, combined with neurohumoral hyperactivation, disrupts homeostatic mechanisms, pushing the condition toward more severe stages with serious consequences.

Despite advances in understanding mitochondria’s role in heart failure, further research is essential to deepen insights into mitochondrial function and its involvement in the disease. Such research could pave the way for more effective treatment strategies. The prospect of targeting mitochondria therapeutically in heart failure patients is promising, as it offers an opportunity to “protect the fort” and prevent further deterioration [23].

## Data Availability

Not applicable.
